# Low-Cost Antenna-Array-Based Metamaterials for Non-Invasive Early-Stage Breast Tumor Detection in the Human Body

**DOI:** 10.3390/bios12100828

**Published:** 2022-10-05

**Authors:** Musa N. Hamza, Yadgar I. Abdulkarim, Salah Raza Saeed, Olcay Altıntaş, Rashad H. Mahmud, Bhargav Appasani, Cristian Ravariu

**Affiliations:** 1 Medical Physics Department, College of Medicals & Applied Science, Charmo University, Chamchamal 46023, Iraq; 2 Department of Physics, College of Science, University of Raparin, Sulaymaniyah 46012, Iraq; 3 Physics Department, College of Science, University of Sulaimani, Sulaimani 46001, Iraq; 4 Department of Electrical-Electronics Engineering, Iskenderun Technical University, Hatay 31200, Turkey; 5 Physics Department, College of Education, Salahaddin University, Erbil 44002, Iraq; 6 School of Electronics Engineering, KIIT University, Bhubaneswar, Odisha 751042, India; 7 Department of Electronic Devices, Circuits and Architectures, Polytechnic University of Bucharest, 060042 Bucharest, Romania

**Keywords:** antenna, artificial magnetic conductor (AMC), breast tumor microwave imaging (MWI), biosensors

## Abstract

Microstrip patch antennas have been used in many applications since their appearance. Despite their great promise, their narrow bandwidth and the loss at high-frequency bands have limited their usage in medical applications. This work proposes a developed low-cost microstrip patch antenna suitable for microwave imaging (MWI) applications within the wideband frequency range. The proposed antenna is loaded with an artificial magnetic conductor (AMC) to improve the antenna performance. The simulated results obtained using computer simulation technology (CST) indicate that the presence of the AMC has improved the frequency selectivity of the antenna at 8.6 GHz with a peak realized gain of 9.90443 dBi and 10.61 dBi for simulated and measured results, respectively. The proposed microstrip antenna has been fabricated to validate the simulated results, and its performance is tested experimentally. Additionally, the fidelity factor of face-to-face (FtF) and side-by-side (SbS) scenarios have been presented. The breast phantom models with a tumor and the antenna operating as a transceiver have been numerically simulated for the application of cancer tumor cell detection. The work will have a significant impact on the design of electromagnetic biosensors.

## 1. Introduction

Cancer describes abnormal cells in the human body that can infect other normal body parts. Breast cancer is the most common cancer in women worldwide, other than non-melanoma skin cancer [1,2]. In 2017 alone, more than 250,000 new instances of breast cancer were diagnosed in the United States. American women will develop breast cancer at a rate of 12% over their lifetime [2]. In 2018, approximately 2.1 million women were diagnosed with breast cancer, of which 626,679 died. The rate is increasing rapidly, with one person diagnosed with breast cancer every 18 s. From 641,000 cases in 1980 to more than 1.6 million cases in 2010, the annual increase in breast cancer incidence was 3.1% [1]. Approximately 8.2 million people have died from breast cancer [3]. Unfortunately, the incidence of breast cancer is expected to rise from 14 million to 22 million people in the next two decades, and it is expected to increase even more [4,5]. More than 1.8 million new breast cancer cases are reported worldwide each year. Due to its high incidence, it is considered one of the most dangerous types of cancer, especially among women, which is why it is the leading cause of death for women worldwide. It results from malignant cells in breast tissue [6,7,8]. More than 40,000 women have died of breast cancer, and 260,000 new breast cancer patients have been identified in the United States [9]. In [9], it is also reported that approximately 500 men have died of breast cancer and the number of new patients among men is 2700. Because of lack of information and awareness among men, the breast cancer death rate in men is higher if we compare it to women. The early diagnosis of breast cancer is considered as the most effective solution to remedy breast cancer [9]. Survival rates can reach 97% when the detection of breast cancer is in the early stage. This ratio demands that we find a new, reliable, and highly efficient way to detect breast cancer at an early stage [6,10].

Mammography via X-ray, magnetic resonance imaging (MRI), and ultrasound are the most common methods physicians select to diagnose breast cancer [11,12]. Mammography is considered the only method for women with no early symptoms. However, many misdiagnoses and inaccurate results have been obtained by these methods, which has caused the deterioration of the patients’ lives [9]. The ionizing nature of X-rays and the pain experienced by women due to the pressuring of their breasts for imagining discourage them from regular checkups [6,8,13]. In ultrasound imaging, the quality of the images is so low that they cannot clearly distinguish between a normal cell and a malignant cell in its early stage [8,9,13]. MRI is a more sensitive technique that can be used for women with dense breasts because of its high sensitivity. However, it is very expensive. In addition, the breast cannot be positioned correctly in this technique, which may lead to an incorrect diagnosis [5,6,13]. The aforementioned techniques have often resulted in errors and limitations in testing, prompting researchers to consider a new technique using microwaves [8,11,13]. Microwave imaging (MWI) systems are currently receiving significant attention as an alternative approach to detecting breast cancer. The main advantages of MWI are the low cost, high data rate accuracy, reduced complexity, non-ionizing nature, comfortable positioning, and very low power density [7].

The basic principle of the MWI technique is to analyze and distinguish between changes in the backscattered signal and changes in the properties of different electrical properties of cells and tissues [6,12,13]. Microwave breast cancer screening techniques can easily distinguish between normal and cancerous cells. This is because the MWI technique relies on changes in the backscattered signal due to changes in the electrical properties of tissues [13].

An antenna is considered an important component of the MWI system. It generates microwave electromagnetic signals to expose the human cells. When the antenna transmits the microwave signal into a human cell, part of the signal scatters back to the receiver depending on the dielectric quality value of the cell. It is important to mention that the dielectric quality of a normal cell is smaller than a malignant cell [5,14]. Thus, a strong backscattered microwave signal clearly indicates a malignant cell. Additionally, further information can be extracted from the scattered back signals that can be used for medical diagnostic purposes [11,13].

A microstrip patch antenna is considered a good candidate to fulfill most of the requirements of the MWI system. However, the main drawbacks are its poor radiation capacity and limited gain (<5 dBi). Metal-backed artificial magnetic conductors (AMCs), unit cell antennas, metamaterial antennas (MTMs), cross-Vivaldi antennas, and slot-loaded antennas [5,6,7,8] are the techniques that are used to improve the microstrip antenna gain, guidance characteristics, and bandwidth. Metamaterial (MTM) is an electromagnetic compound with very different properties that are rarely encountered in nature. Recently, a new era in MWI has been ushered in by developments of MTMs with immense potential for deploying MWI devices and negative-scale MTM applications. The antennas designed for microwave imaging will be based on the properties of MTMs, possessing external qualities such as permeability, permittivity, wavelength, wave radiation control, etc. Special properties of MTMs have led researchers to use them in designing microstrip antennas for many medical applications, especially in detecting abnormal cells and breast cancer. Additionally, the artificial magnetic conductor (AMC), also known as phase reflection, is another type of artificial structure that is periodically loaded and exploited to reduce backscattered radiation or limit the propagation of antennas in a particular direction. In addition, it is a potential way to further improve the performance of antennas in terms of radiation, directionality, gain, and bandwidth. A synthetic magnetic conductor that does not exist in nature is the cause of this phase reflection [5,6,8].

MTMs are man-made objects that display unusual characteristics such as negative permittivity (ε < 0), negative permeability (µ < 0), and negative refractive index [15] in the desired frequency range [16,17]. They are used in a variety of technical applications, including sensors [17,18,19], especially in the microwave and terahertz regions [5,17,20,21], antennas [5,22,23,24,25], sensitive detectors [20], radar [26,27], polarization transducers [27,28], cloaking [29], and absorbers [17,27,30]. Experimentally, Landy proposed the concept of a perfect MTM absorber in 2008 [31]. Since then, other single bands [32], the dual band [33], the multi band [34], and broadband absorbers made of MTMs have been introduced [35]. In this paper, we have numerically and experimentally presented a new microstrip patch antenna design by incorporating an artificial magnetic conductor for breast cancer detection using microwave imaging. CST software was used to design and simulate numerical results. The proposed design was manufactured by using an LPKF PCB prototyping machine and the experimental studies were conducted by the Agilent PNA-L vector network analyzer (VNA) to measure the recommended structure’s return loss, gain, and radiation pattern results. Before the measurements, the VNA was calibrated by an open circuit, short circuit, and 50-ohm load element in the desired frequency range. The proposed antenna has a peak realized gain of 10.61 dBi according to test results. The radiation pattern, the fidelity factor of face-to-face (FtF) and side-by-side (SbS) scenarios, group delay, and field distributions have been presented in this work. The novelty of the proposed structure can be emphasized by its simple, low-profile design. Interestingly, with the collection of these good features, the proposed structure can be used for both detection and imaging; furthermore, the gain for this work is 10.61 dBi, which is superior compared to that of the structures reported in the literature [5,6,7,12,13,36]. In addition, the direction of the radiation pattern is very important for working in the detection of breast cancer cells. All previous studies that have used Vivaldi antennas within our scope have directed their radiation patterns from the top of the antenna, which causes many problems when joining the antenna to the metamaterial and requires a large distance between them, which increases the error rate. However, in this work the radiation pattern comes out directly from the middle of the antenna and the distance between the antenna and the AMC is very small. We believe this study is useful for medical applications such as cancer tumor cell detection.

## 2. Metamaterial Unit Cell and AMC Design Layout

**Figure 1:**
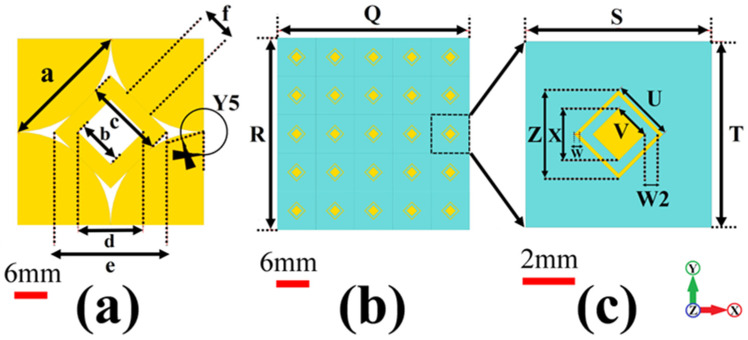
(**a**) AMC structure geometric design rear view, (**b**) AMC structure geometric design front view, (**c**) MTM unit cell geometric design.

The unit cell of the MTM proposed for the microstrip patch antenna is shown in Figure 1b. It consists of two concentric rhombuses made of copper. The dimensions of these concentric rhombuses are shown in Table 1. The AMC consists of a 5 × 5 array of these concentric rhombuses on top of an FR4 substrate (flame retardant type 4) due to its low cost and the fact that it provides adequate electrical insulation and high mechanical strength, with a bottom defected ground structure. The bottom defected ground structure is shown in Figure 1c. The AMC has designed the CST simulator.

## 3. Antenna Sensor Design with MTM

### The Schematic Layout of the Optimized MTM

**Figure 2:**
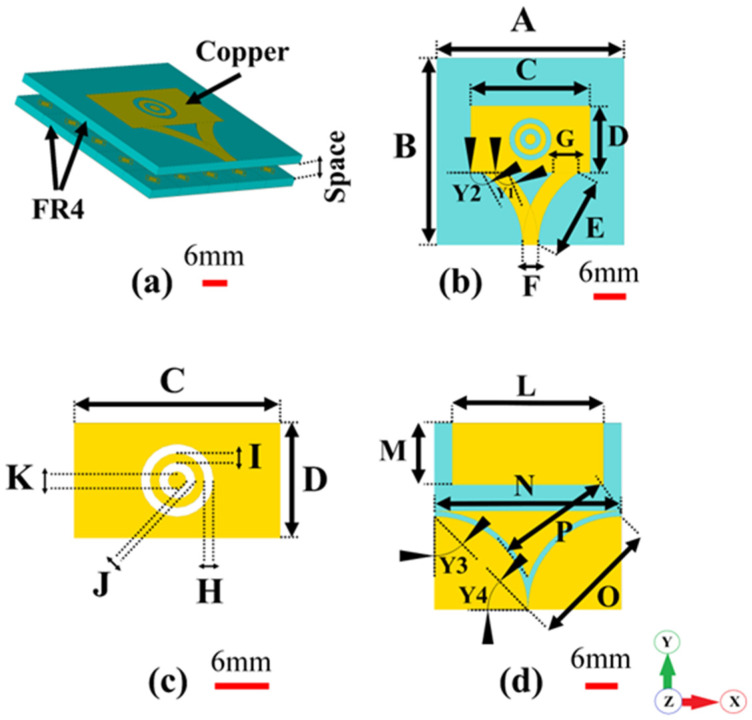
Geometric structure of the proposed microstrip patch antenna: (**a**) perspective view, (**b**) front view, (**c**) microstrip patch, (**d**) backside view.

The design of the proposed antenna-based metamaterial sensor is shown in Figure 2. It is based on the microstrip structure. As shown in Figure 2a, two circular rings etched from the top patch layer are formed to allow the antenna to operate over a large frequency band. Two curved conducting strips integrated with the patch are used to feed the antenna. A defected ground plane with two curved paths is added beneath the substrate, as shown in Figure 2c. The curved stripes and paths are utilized to maintain a large impedance bandwidth for the antenna sensor. All the physical dimensions of the proposed antenna-sensor are summarized in Table 1.

**Figure 3:**
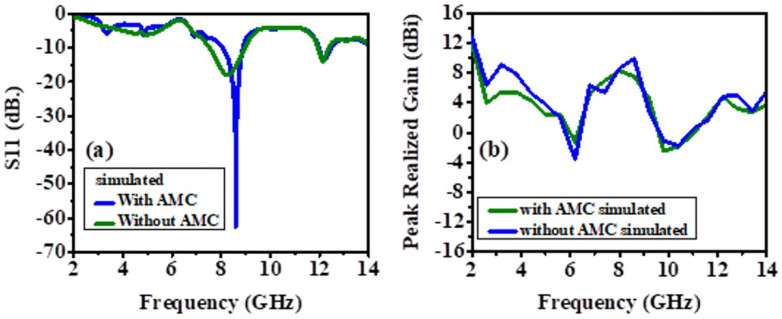
(**a**) The simulated reflection coefficient (S11) AMC and without AMC, (**b**) the simulated maximum gain of the prototype with AMC and without AMC.

The designed AMC has been simulated for its transmission and reflection coefficients, which are shown in Figure 3. The reflection coefficient clearly shows that the designed AMC resonates at 8.6 GHz, thereby improving the radiation characteristics of the antenna that will be placed on top of the AMC. The frequency responses of the antenna-based metamaterial sensor are obtained using the CST simulator. Figure 3a shows the reflection coefficient (S11) with and without the presence of the designed artificial magnetic conductor (AMC) layer. One can depict that the AMC layer has no obvious impact on the S11 response at the start and stop band. However, a better matching around the operating center frequency of 8.6 GHz is achieved when the AMC layer is present. The peak realized gain of the antenna-sensor is shown in Figure 3b. The presence of the AMC layer improves the realized gain around the center frequency (8.6 GHz) by a factor of 2 dBi.

## 4. Results and Discussion

### 4.1. Frequency-Domain Performance

#### 4.1.1. Proposed Antenna Image after Fabrication

**Figure 4:**
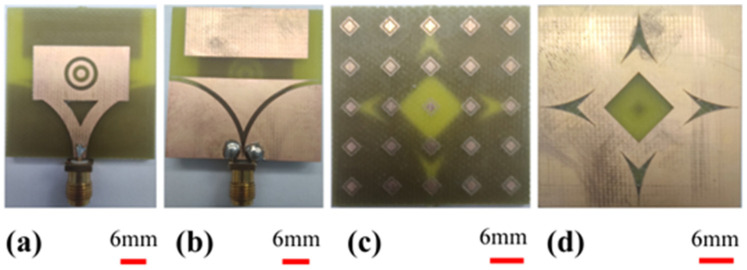
Fabricated prototype: (**a**) microstrip patch antenna front view, (**b**) microstrip patch antenna rear view, (**c**) AMC structure front view, (**d**) AMC structure rear view.

The antenna and AMC structure prototypes are manufactured using an LPKF PCB prototyping machine and the structures are illustrated in Figure 4. As in the numerical studies, the FR4-type dielectric layers have been used to obtain the proposed structure. The antenna’s patch side has complementary two nested circular rings. The background of the antenna is inspired by the Vivaldi shape as in Figure 4b. The top side of the AMC structure consists of 5 × 5 unit cells in Figure 4c. The bottom side of it has a square space with four arrow-type notches, demonstrated in Figure 4d.

**Figure 5:**
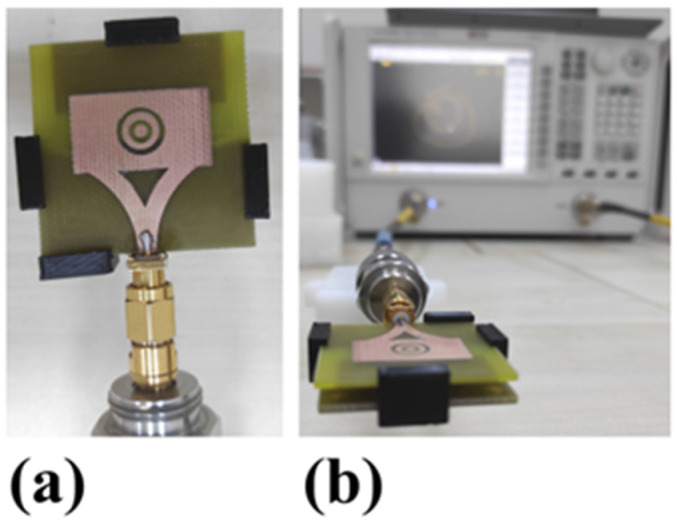
(**a**) Fabricated proposed design and (**b**) experimental measured set-up showed combined antenna structure with vector network analyzer (VNA).

Experimental studies have been conducted by the Agilent PNA-L vector network analyzer (VNA, Keysight company, Colorado Springs, CO, USA) to measure the proposed integrated structure’s return loss, gain, and radiation pattern results. Before the measurements, the VNA was calibrated by an open circuit, short circuit, and 50-ohm load element between 6 GHz and 10 GHz. Furthermore, four handles fabricated by a 3D printer have been placed between the antenna and AMC structure to fix the right distance, as in the numerical studies illustrated in Figure 5.

#### 4.1.2. Return Loss (S11) and Realized Gain

**Figure 6:**
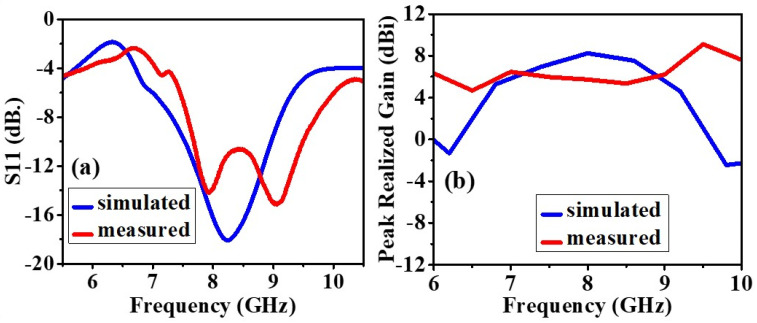
Without AMC: (**a**) The measured and simulated reflection coefficient (S11), (**b**) The measured and simulated maximum gain of the prototype.

**Figure 7:**
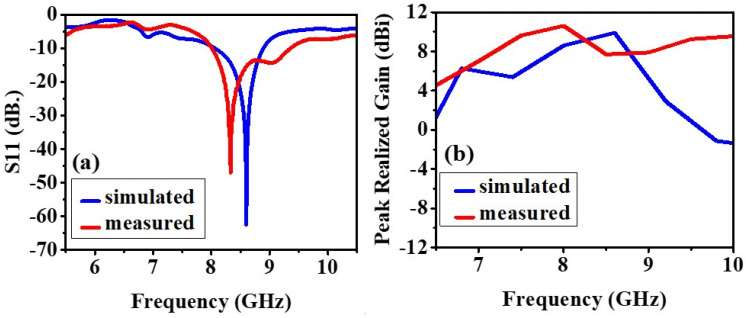
With AMC: (**a**) The measured and simulated reflection coefficient (S11), (**b**) The measured and simulated maximum gain of the prototype.

The experimental and simulated results of the antenna without the AMC backing are shown in Figure 6 and Figure 7. The slight difference in the results is due to the fabrication defects and the use of soldering to feed the antenna with an SMA connector. This section compares antenna performance with and without the AMC structure concerning the return loss and realized gain parameters. In the analysis of the antenna without the AMC structure, the return losses at the lowest peaks have been monitored at 8.24 GHz with 18.09 dB for simulated results and around 7.905 GHz with 14.138 dB, and 9.02 GHz with 15.124 dB for measured results, illustrated in Figure 6a. When the AMC is placed below the antenna, the return loss is further minimized, and the radiation is significantly improved, and these results are shown in Figure 7a. As to the analysis of the antenna with the AMC structure, this result has been observed as 8.6 GHz with 62.515 dB for the simulated results and 8.33 GHz with 47 dB for the measured results, illustrated in Figure 7a. Although there are small frequency deviations between the simulated and measured results, it can be seen from these results that the AMC structure immensely improves the return loss parameters.

Metallic structures, having flat and smooth face features, possess the ability to support surface waves which creates electromagnetic wave propagation between the metal and free space [37]. When a surface wave at microwave regime faces distortions, bending, or discontinuous surface patterns, it radiates vertically [38]. At this point, the gaps or slots in the ground plane of the proposed antenna given in Figure 2d create discontinuity. Therefore, the surface wave which encounters this discontinuity is vertically propagated to the AMC structure placed in the back side of the antenna with a zero-reflection phase. Next, the AMC reflects the emerging electromagnetic waves. These reflected electromagnetic waves from the AMC constructively integrate into a radiated wave stem from the antenna and this causes it to increase in overall gain [39]. As the proposed AMC structure produces a zero-reflection phase at 8.6 GHz, the overall gain remarkably increases at this frequency point. To provide the null reflection phase property, it is important to obtain impedance matching between the AMC and the antenna. In this application, the space distance between the AMC and the antenna given in Table 1 was chosen as 2.8 mm. When the distance is below 2.8 mm, the mutual coupling deteriorates the impedance matching and also the zero-reflection phase property.

Figure 6b and Figure 7b demonstrate the peak realized gain of the antennas with and without AMC structure. The realized gains without AMC structure at the resonance frequencies (return losses at the lowest value) are 8.24617 dBi and 9.12 dBi for simulated and measured results, respectively. These results have been obtained as 9.90443 dBi and 10.61 dBi for simulated and measured results, respectively. The results for both of them after the AMC structure is integrated into the antenna can be seen in Figure 7b. Hence, it can be also concluded that the AMC structure plays an important role in enhancing the antenna’s gain.

#### 4.1.3. Radiation Pattern

**Figure 8:**
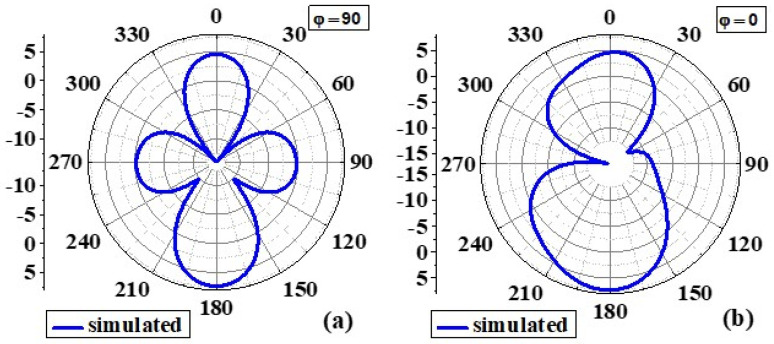
Simulated 2D radiation pattern of the proposed antenna without AMC at 8.6GHz: (**a**) φ = 90 and (**b**) φ = 0.

**Figure 9:**
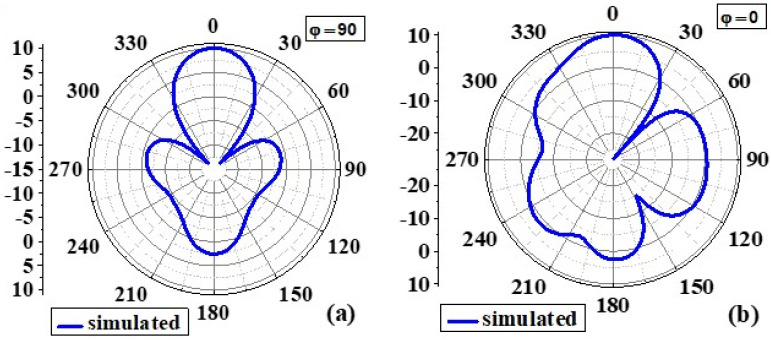
Simulated 2D radiation pattern of the proposed antenna with AMC at 8.6GHz: (**a**) φ = 90 and (**b**) φ = 0.

**Figure 10:**
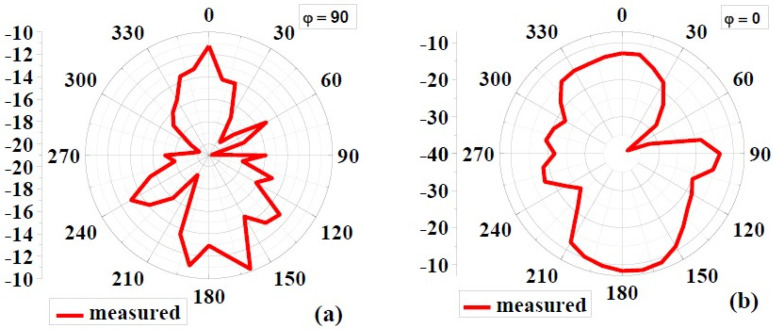
Measured 2D radiation pattern of the proposed antenna without AMC at 8.6GHz: (**a**) φ = 90 and (**b**) φ = 0.

**Figure 11:**
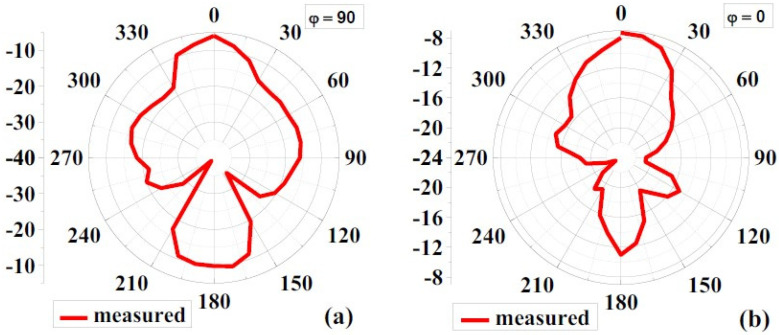
Measured 2D radiation pattern of the proposed antenna with AMC at 8.6GHz: (**a**) φ = 90, (**b**) φ = 0.

Figure 8 and Figure 9 show the simulated 2D radiation patterns of antennas without the AMC configuration and antennas with the AMC configuration in the xz (phi 0) and yz (phi 90) planes, respectively. As previously described, the AMC configuration reduced dorsal and focused radiation in a broad lateral direction. Compared to an antenna without an AMC structure, the antenna with an AMC structure exhibited more directed radiation. Strength is enhanced along the side of the bore using the proposed AMC. The structure lowers surface waves. This shows how the use of the AMC enhances the radiation characteristics such as gain, directionality, reflection coefficient (S11), etc. [6]. Figure 10 and Figure 11 show measured 2D radiation patterns for resonant frequencies in the principal planes. The antenna is measured in a lab using theta stepping and rolling on the phi axis. Theta and phi spherical coordinate tables record the measurements. The following is how the spherical coordinates relate to the Cartesian axes: Theta = 0 for 360 and Phi = 0 for XZ Cut, Phi = 90 for YZ Cut, and Theta = 90 for XY Cut. The xz plane (φ = 0) and the yz plane (φ = 90) are referred to as the E and H planes, respectively [6,40].

The near-field efficiency shows that the antenna became unidirectional when the AMC destination configuration was used. E-plane and H-plane beams are preferred because the orientation of the primary lobes is stable with broadside beams. The directionality of the antenna was, therefore, improved. Another notable feature is the introduction of a very small rear lobe. Because of the higher order excitation, radiation modes at higher frequencies, particularly those above 6 GHz, have fewer nulls. According to the debate, an antenna with an AMC component has the lowest energy loss compared to an antenna without an AMC due to its reflection quality [6,41].

### 4.2. Time-Domain Performance

Another characteristic to evaluate the good performance of the antenna in the time domain is determining the accuracy of the proposed antenna, especially in microwave imaging systems. Indeed, although the proposed antenna has shown excellent frequency-domain results, these characteristics alone are not enough criteria to ensure good antenna performance in the time domain. Therefore, to obtain the time-domain study and ensure the antenna’s excellent performance, two identical antennas are installed in two different directions, so that the second antenna receives the signal sent by the first antenna. Consequently, the input–output pulse, time-domain efficiency, transmission coefficient (S_21_), and group delay (τ) can be evaluated to ensure the good performance of the antenna in the time domain [5,13,36].

#### 4.2.1. Fidelity Factor (FF) Input and Received Pulse Waveforms (in a Face-to-Face and Side-by-Side Situation)

**Figure 12:**
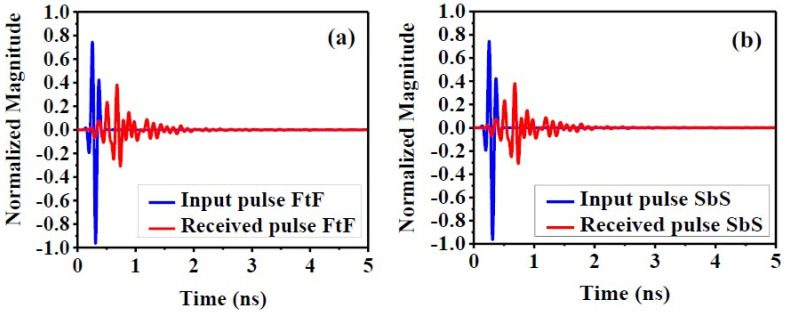
Normalized transmitted and received pulse in: (**a**) face-to-face, (**b**) side-by-side.

The RX and TX pulse graphs show that the SbS and FtF directions have little attenuation in signal reception. In addition, between the transmitted and received signal, the maximum magnitude of the cross-correlation between them identifies the fidelity factor. What is mentioned in Equation (1) can be determined [5,13,36]. Figure 12a,b shows both the input signal and the received signal so that the spacing between two proposed homogeneous antennas is 200 mm and both SbS and FtF directions are shown.

(1) $$F=Max_{\tau }\left|\frac{\int_{-\infty }^{+\infty }s\left(t\right)r\left(t-\tau \right)}{\int_{-\infty }^{+\infty }s{\left(t\right)}^{2}dt\int_{-\infty }^{+\infty }r{\left(t\right)}^{2}dt}\right|$$

where, respectively, *s(t)* and *r(t)* stand in for the transmitter (TX) and received (RX) signals.

#### 4.2.2. Group Delay (in Face-to-Face and Side-by-Side Scenarios)

**Figure 13:**
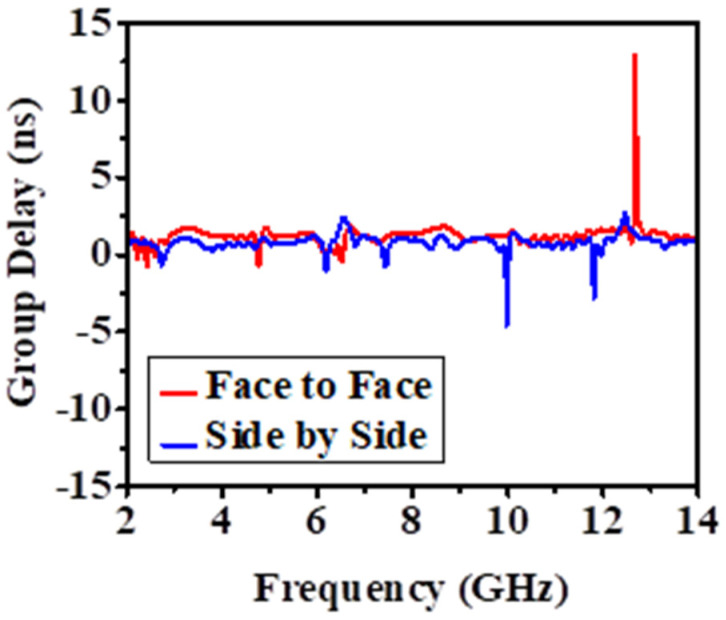
The group delay variation for Face-to-Face (FtF) and Side-by-Side (SbS).

The difference (*τ*) as a function of frequency for both SbS and FtF directions is shown and explained in Figure 13. The group delay (*τ*) can be defined as the time delay required for signals to propagate from the transmitting end to the receiving end. Equation (2) explains the determination of the group delay (*τ*) [5,36].

(2) $$\tau =-\frac{d\theta \left(\omega \right)}{d\left(\omega \right)}\;$$

where the signal phase (*θ*) and frequency (*ω*) are represented by the units (in radians) and (in radians/sec), respectively.

Remember that the antennas are 200 mm apart and that (*τ*) is simulated for both directions. The antenna may broadcast short pulses with ringing and little late-time distortion due to the delay plot of the group in the practical linear FtF distribution. As Figure 13 shows very clearly, it is advisable to use the proposed antenna in the FtF direction.

## 5. Microwave Imaging Setup and Measurement

### 5.1. Microwave Imaging Setup (Breast Phantom and Antenna Array Setup Surrounding Phantom)

**Figure 14:**
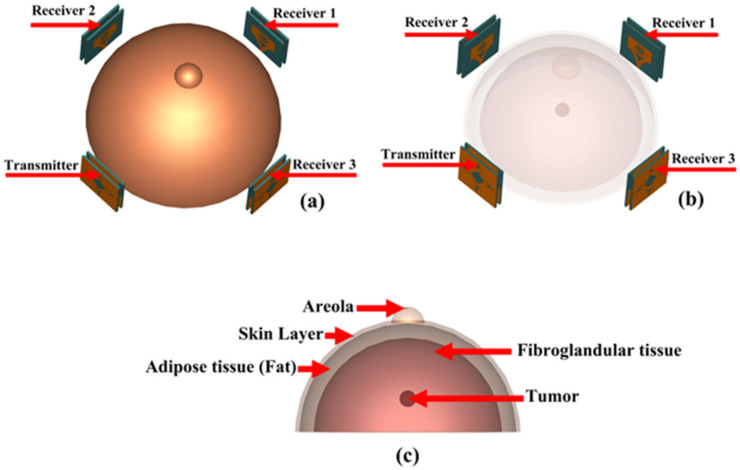
(**a**) and (**b**) simulation setup for MWI, (**c**) breast phantom.

The performance of the antenna in the frequency domain and time domain indicates that it can be used as the radiating element for microwave imaging of the breast and detecting tumors. Four antennas are placed surrounding the breast to detect breast tumors, as shown in Figure 14. The breast model is constructed as a five-layered structure. The tumor in the breast can be benign or malignant. In fact, the cure rate after the metastatic stage of breast cancer cells is very low and the survival rate is dire. Therefore, we have focused very intensively on detection at the earliest stage because the pass rate is more than 90% at this stage. However, if we have more than one tumor it means that the patient is out of the early stage and the cancer cells have grown and spread to the surrounding cells, which is not the goal we are focusing on. The properties of these various layers are shown in Table 2.

### 5.2. S-Parameters of The Antenna Array (with Only One Malignant Cell, with Tumor and Normal Cells)

**Figure 15:**
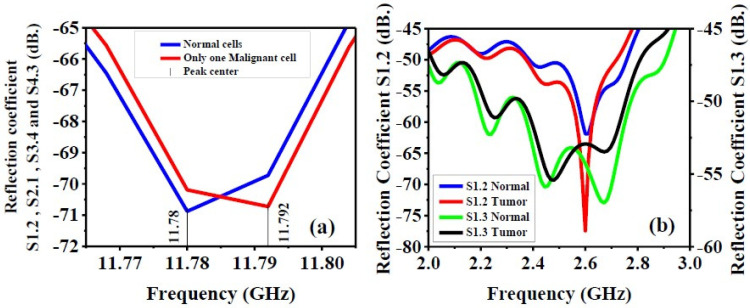
(**a**) Reflection coefficient with the presence of only one malignant cell inside the breast phantom. (**b**) Reflection coefficient (S-parameters) (S1.2 and S1.3) with the presence of tumor inside breast phantom.

This section exhibits the influence of a tumor inside the breast phantom on the scattering parameters of the antenna array. Figure 15a shows the S1.2 and S3.4 responses versus frequencies. It can be depicted that the presence of a single malignant cell causes an obvious shift of the peak center scattering response. On the other hand, the impact of a tumor on the antenna reflection coefficients (S1.2, S1.3) is shown in Figure 15b. With the presence of a tumor, a better matching and more reflections occur over the whole operating frequency range.

**Figure 16:**
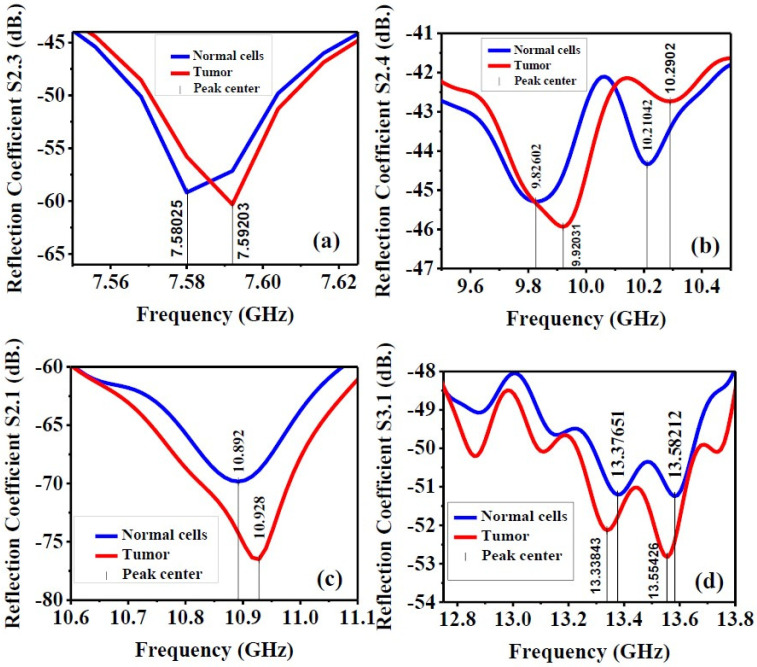
Reflection coefficient with the presence of tumor inside breast phantom: (**a**) S2.3, (**b**) S2.4, (**c**) S2.1, (**d**) S3.1.

Figure 16 indicates that the presence of a tumor in a cell not only shifts the peak center of the scattering parameters to the higher operating frequencies when compared with a normal cell, but also has more significant reflections. For instance, in Figure 16a, the S2.3 peak center shifts from 7.58025 GHz to 7.59203 GHz, with a larger reflection value due to the tumor. Similar behavior can be noticed in the S2.4 parameter, as shown in Figure 16b, except at higher operating frequencies, the S2.4 value for a cell with a tumor is less than a normal cell. Although the frequency shifts in the S2.1 and S3.1 responses are not as large as the ones obtained in the S2.3 and S2.4 responses, stronger reflections of the presence of the tumor are expected. This could be extremely useful in detecting a tumor. In the next steps, we hope that we are able to chemically fabricate the breast phantom to have the same dielectric properties as a real human breast, in order to deploy the fabricated antenna and experimentally measure it.

### 5.3. Microwave Imaging (MWI) Results

**Figure 17:**
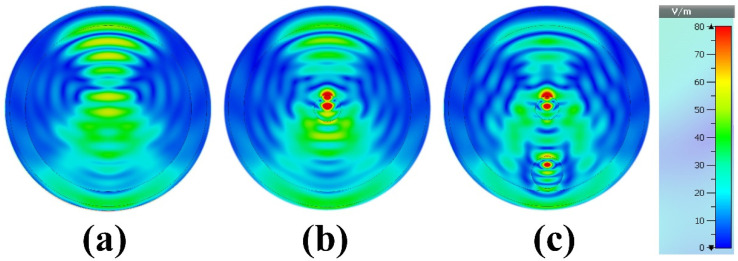
Reconstructed images of the breast phantom E-field (microwave imaging results): (**a**) phantom without tumor, (**b**) phantom with one tumor and (**c**) phantom with two tumors at 8.6 GHz.

**Figure 18:**
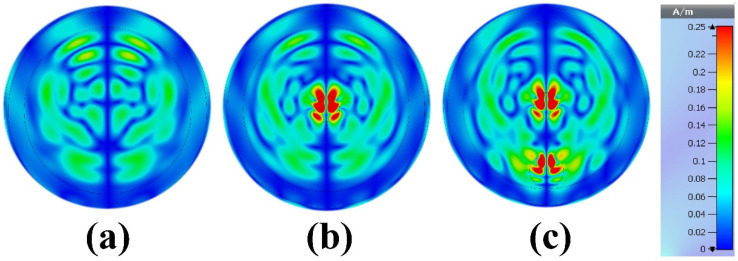
Reconstructed images of the breast phantom (H-field microwave imaging results): (**a**) phantom without tumor, (**b**) phantom with one tumor and (**c**) phantom with two tumors at 8.6 GHz.

MWI of the breast phantom is obtained from simulation results by plotting the electric field distribution at 8.6 GHz. The MWI results are shown in Figure 17 and Figure 18. Only the first antenna, the transmission characteristics of which were discussed earlier, is excited. We conducted breast phantom scanning for three different cases, so that in the first case the breast phantom is without a tumor, in the second case the breast phantom contains one tumor equal to 5 mm in radius, located in the middle of the breast phantom, meaning it is fibroglandular in nature. However, in the third case, we added another tumor, i.e., the breast phantom contains two tumors, the radius of the second tumor is equal to 6 mm, the location is different from the first tumor. One of the tumors is located in fibroglandular tissue and the other is located in adipose tissue (fat), and the distance between the first tumor and the second tumor from base to base is equal to 20 mm horizontally. The presence of a tumor increases the concentration of the electric field compared to the tissue without the tumor. This can help in diagnosing the presence of the tumor but also in locating its position inside the breast. It is examined that the target tumor has been visibly detected with a red color. However, it is noticeable that our proposed system can be a decent candidate for microwave breast imaging to identify the tumor by examining the backscattering signals proficiently.

Figure 18 shows the color map magnetic field distribution for the reconstruction phantom without tumor, with one tumor and with two tumors at the resonances frequency of 8.6 GHz. As we can see from Figure 18a, the magnetic field is more localized around the phantom when there is no tumor, the magnetic field is distributed at the center of the phantom when there is a tumor, and the magnetic field is distributed around the phantom when there are two tumors. The proposed MWI system has some advantages over other published articles in terms of size, compactness, and cost-effectiveness. The proposed design exhibits better performance for the breast phantom, which is promising for early-stage tumor detection.

### 5.4. Microwave Imaging System (MIS)

**Figure 19:**
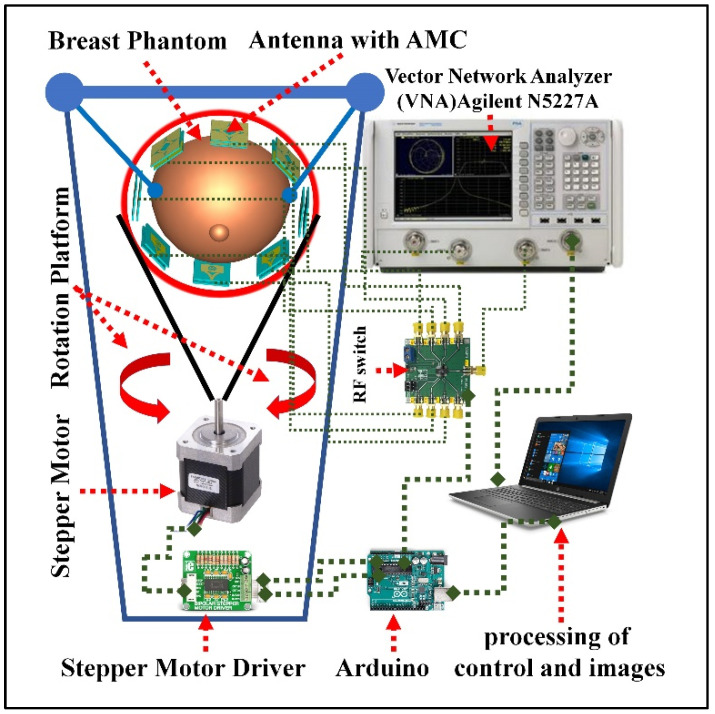
The breast imaging system presented is shown in schematic form with its many elements.

We can summarize the microwave imaging system as explained in Figure 19 so that it shows the architecture and different parts of the proposed imaging system for the breast phantom. The designed MIS consists of eight antenna arrays, where one antenna acts as a transmitter and the other seven antennas receive scattered signals, an 8-port RF converter system to control the receivers, a convenient suspension platform of the breast phantom, and a MATLAB-based laptop computer program for signal processing and image reconstruction of the different components of the breast phantom on the basis of the scattered signals data collected by the receiving antennas. The antennas should be arranged for this application on a transparent, circular ABS plastic container so that it is suitable for rotation around the breast phantom while scanning. The plastic container should be mounted on a stepper motor with an SD02B controller. During the scanning, the suspension platform is used to place the phantom in the antenna array; this arrangement should be such that the distance between the placed antenna and the breast phantom is kept at 2 cm. It is very important to be able to change the angle of the antennas during scanning in order to obtain excellent results and high-quality images, so the step motor on the mechanical rotation platform should be able to rotate the array in polar coordinates from 0 to 180 degrees. Using a special type of wire known as a coaxial wire, all antennas are connected to the RF switch. Microwave signals in the frequency domain to be dedicated for this type of scanning are produced and sent to the breast phantom through port 1 of the VNA. The backscattered data (S21, S31, S71) are collected via VNA (Agilent N5227A, Keysight, Colorado Springs, CO, USA) and MATLAB program on PC. In general, the abundance and deficit of received data depends on the number of antennas, the number of scans, and the scanning angle of the antennas with the breast phantom.

### 5.5. Surface Current Distribution

**Figure 20:**
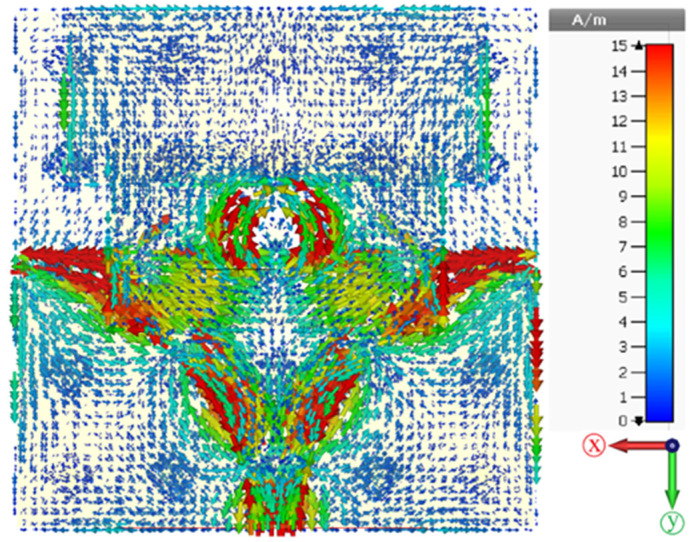
Surface current distribution at 8.6 GHz.

Figure 20 shows the surface current distribution for the proposed antenna-loaded artificial magnetic conductor. As we can see from the figure below, the surface current flows from the two microstrips and distribution for both sides also flows in a parallel and anti-parallel direction around the circular patch, in agreement with other methods [44].

Table 3 shows the obtained results of the proposed design compared with other reported antenna-based metamaterial sensors in terms of overall structure, size, substrate, operating frequency, gain, and published year. Results show that the proposed structure outperformed those reported in the literature.

## 6. Conclusions

In summary, a new structure and low-cost antenna were designed, fabricated, and tested for microwave breast imaging at 2–14 GHz frequencies. CST software was used to design and simulate the structure; the antenna and AMC structure prototypes were manufactured using an LPKF PCB prototyping machine. The realized gains without the AMC structure at the resonance frequencies (return losses at the lowest value) were 8.24617 dBi and 9.12 dBi for simulated and measured results, respectively, while with the AMC integrated into the antenna, the gains improved by 9.90443 dBi and 10.61 dBi for simulated and measured results, respectively. The fidelity factor of face-to-face (FtF) and side-by-side (SbS) scenarios were investigated. Phantom models with and without tumors were considered to validate the approach. The proposed design is a good candidate for biosensor applications and microwave breast imaging.

## Figures and Tables

**Table 1:** All optimized parameters of the proposed prototype.

Parameter	Value (mm)	Parameter	Value (mm)	Parameter	Value (mm)	Parameter	Value (mm)
A	35.60	K	2.00	U	2.40	a	25.17
B	35.60	L	28.80	V	1.41	b	8.77
C	22.70	M	11.80	W	0.20	c	15.27
D	12.70	N	35.60	X	2.00	d	12.40
E	15.77	O	25.17	Y1	112.82°	e	21.60
F	3.00	P	21.90	Y2	118.92°	f	6.11
G	4.82	Q	35.60	Y3	45.00°	Space	2.8
H	1.00	R	35.60	Y4	45.00°	W2	0.500
I	1.00	S	7.12	Y5	287.56°		
J	1.00	T	7.12	Z	3.40		

**Table 2:** The dielectric properties of the human breast tissues with tumor and malignant cells.

Tissue Type	Effective Dielectric Permittivity (εeff) (F/m) [5,8,42]	Electric or Effective Conductivity (σ eff) (S/m) [5,8,42]	Density (kg/m^3^) [5,8]	Thermal Conductivity *K* (W/(K·m)) [43]	Mu (µ)	Specific Heat Capacity *Cp* (kJ/(K·kg)) [43]	Diffusivity (m^2^/s)
Areola	36.7	2.34	1109	0.52	1	3.92	1.19615 × 10^−7^
Skin	36.7	2.34	1109	0.52	1	3.92	1.19615 × 10^−7^
Adipose tissue (fat)	4.84	0.262	911	0.23	1	1.9	1.32879 × 10^−7^
Fibroglandular	20.1	0.5	1035	0.51	1	3.9	1.26347 × 10^−7^
Tumor	67	4	1085	0.55	1	3.75	1.35177 × 10^−7^
Malignant cell	15.12	2.346	1085	0.55	1	3.75	1.35177 × 10^−7^

**Table 3:** Comparison between the proposed and existing works in terms of various features.

References	Structure Size (mm^2^)	Substrate	Frequency Range (GHz)	Gain (dBi)	Year Published
[5]	20 × 19	FR4	2–12	5	2022
[6]	66 × 66	Rogers RO4003C	1–13	10	2018
[7]	51 × 42	Rogers RT/duroid 5870	2–7.5	9.5	2019
[13]	42 × 41	Rogers RT 5880	2–11	5.48	2020
[12]	80 × 61	felt	4–15	7.56	2022
[36]	21.44 × 23.53	FR4	3–12	5.76	2019
This work	35.6 × 35.6	FR4	2–14	10.61	2022

## Data Availability

Not applicable.
